# The factors affecting implementing shared decision-making in clinical trials: a cross-sectional survey of clinical research coordinators’ perceptions in Japan

**DOI:** 10.1186/s12911-023-02138-y

**Published:** 2023-02-23

**Authors:** Miho Fujita, Yuki Yonekura, Kazuhiro Nakayama

**Affiliations:** 1 Clinical Research Support Office, Showa University Northern Yokohama Hospital, 35-1 Chigasaki-chuo, Tsuzuki-ku, Yokohama-shi, 224-8501 Japan; 2 Graduate School of Nursing Science, St. Luke’s International University, Tokyo, Japan

**Keywords:** Shared decision-making, Implementation, Clinical trial, Clinical research coordinators’ perception, The Theory of Planned behaviour, SDM-Q-Doc, Survey

## Abstract

**Background:**

The shared decision-making model has been proposed as the ideal treatment decision-making process in medical encounters. However, the decision to participate in clinical trials rarely involves shared decision-making. In this study, we investigated the perceptions of Japanese clinical research coordinators who routinely support the informed consent process.

**Methods:**

This study aimed to (1) identify clinical research coordinators’ perceptions of the current status of shared decision-making implementation and its influencing factors, and (2) obtain suggestions to enhance the shared decision-making process in clinical trials. A cross-sectional survey was conducted using a web questionnaire based on the Theory of Planned behaviour. Invitations were sent to 1087 Japanese medical institutions, and responses from the participants were captured via the web. The shared decision-making process in clinical trials was defined according to the Shared Decision-Making Questionnaire for Doctors. The effect of the attitudes toward shared decision-making, clinical research coordinators’ subjective norms towards its implementation, perceived barriers to autonomous decision-making, and the number of difficult steps in the shared decision-making process on the shared decision-making current status as the shared decision-making intention was assessed by multiple regression analysis.

**Results:**

In total, 373 clinical research coordinators responded to the questionnaire. Many believed that they were already implementing shared decision-making. Attitudes toward shared decision-making (*t* = 3.400, *p* < .001), clinical research coordinators’ subjective norms towards its implementation (*t* = 2.239, *p* = .026), perceived barriers to autonomous decision-making (*t* = 3.957, *p* < .001), and the number of difficult steps in the shared decision-making process (*t* = 3.317, *p* = .001) were found to significantly influence current status (Adjusted *R*^2^ = .123). However, results on perceived barriers to autonomous decision-making and the number of difficult steps in the shared decision-making process indicate a lack of knowledge of shared decision-making and decision-support skills among clinical research coordinators.

**Conclusions:**

Clinical research coordinators might positively perceive shared decision-making based on normative beliefs without sufficient knowledge of it. Therefore, providing appropriate training on shared decision-making to clinical research coordinators and increasing awareness among stakeholders could enable its improvement.

***Trial registration*:**

Not applicable.

## Background

Clinical medicine aims to provide optimal medical care for individual patients, while clinical trials aim to answer scientific questions to produce generalisable knowledge that will benefit future patients [1]. Therefore, obtaining informed consent (IC) to conduct clinical trials is a crucial component of protecting patients’ human rights; IC is intended to protect and enable patients to make autonomous or self-determined choices. Furthermore, patient participation in decision-making has evolved from IC to include broader principles of patient autonomy, control, and challenging the authority of physicians [2].

Shared decision-making (SDM) has been proposed as the ideal model of treatment decision-making in the medical encounter [2] and is considered the pinnacle of patient-centred care [3]. The theoretical key features of SDM include (1) at least two parties (patient and physician) are involved, (2) information is exchanged, (3) both parties are aware of treatment options, and (4) both parties bring their decision criteria actively and equally into the decision-making process [2, 4]. Stiggelbout et al. outlined four elements of the SDM process, whereby a healthcare professional: (1) informs the patient that a decision must be made and that their opinion is important; (2) explains the pros and cons of available options; (3) discusses options and helps patients make decisions based on their preferences; (4) discusses the patient’s choice, or defers the decision, and schedules a follow-up [5].

The overlaps and differences between IC and SDM in clinical trials are unclear. However, medical ethicists have argued that SDM respects a patient’s relational autonomy by recognising it as a capability that depends on interpersonal relationships and broader health care and social conditions [6]. Faden and Beauchamp explain that autonomy requires acting (1) *intentionally*, (2) *with understanding*, and (3) *without controlling influences that determine one’s action* [7]. Relational autonomy relies on social relationships to fulfil its purpose. In other words, SDM complements the challenges of IC based on the traditional concept of autonomy, which is guided by ‘self-governance’ [8], and it can improve the quality of decision-making.

However, the decision to participate in clinical trials rarely involves SDM, and several issues that inhibit respect for autonomy remain. First, many previous studies have noted the difficulty and lack of appropriate understanding of informed consent documents (ICDs). Long, complex ICDs may obscure pertinent information from the potential research participant and appear to be designed primarily to protect institutions and meet regulatory needs, rather than to inform the potential participant [9]. Moreover, systematic reviews found a lack of understanding of the altruistic purpose of clinical trials, the randomisation process, placebos, being a volunteer, the right to withdraw consent, and the benefits and disadvantages of trial participation [10, 11]. Although many researchers have examined interventions to improve ICDs and support understanding, the proportion of participants who understood IC had not increased over 30 years [12].

Second, the influence of the patient-physician or patient-family relationships on decision-making has been noted. In a meta-analysis of qualitative studies on cancer patients’ reasons for participating in clinical trials, trust in physicians and relatives’ attitudes toward clinical trials were cited as influencing factors. Nielsen and Berthelsen argue that these influences may compromise the voluntary principle of the International Conference on Harmonisation of Technical Requirements for Registration of Pharmaceuticals for Human Use Good Clinical Practice [13].

In Japan, clinical research coordinators (CRCs) play important roles in the ethical and scientific implementation of clinical trial procedures. They act as liaisons between investigators and human research subjects, clinical care providers, regulatory bodies, sponsors, and other stakeholders in the research process [14]. In addition, the sponsor bears the site support cost of CRCs; therefore, CRCs are expected to recruit a certain number of patients within a timeframe to conduct studies. According to a 2020 report by the Japan Site Management Organization (JSMO) [15], most CRCs in Japan are nurses, medical laboratory scientists and pharmacists, and other professionals with clinical qualifications; however, approximately 30% of CRCs do not have medical qualifications. Most CRCs receive training through various sources, including the Ministry of Health, Labour and Welfare (MHLW), professional organisations, and the Japan Clinical Research Core Hospital Consortium (J-CCRC). CRCs always receive training before being delegated to clinical trials; thus, they are educated in research ethics, relevant regulations, and target diseases and clinical trial interventions.

In the IC process, CRCs improve ICDs, prepare supplementary materials, and assist investigators in their explanations; however, issues related to respect for autonomy remain unresolved.

For instance, a survey of 376 participants in a randomised controlled trial examining the effectiveness of breast cancer screening by ultrasound showed that the experimental nature of the study, potential risks or discomfort, benefit to self, and compensation were poorly understood [16]. Miyata and Sato conducted semi-structured interviews and questionnaires among 16 participants in clinical trials. They found that even patients who self-reportedly understood the clinical trial’s contents stated that their reasons for participation were ‘treatment of my disease’, ‘trust in my doctor’, and ‘recommendation from my doctor’. Meanwhile, others stated: ‘I thought my disease would worsen if I did not participate in the clinical trial’ [17]. In addition, Fujita analysed the blog posts of patients considering participation in clinical trials and found that some patients changed their decisions because of family members’ attitudes [18].

Hallinan et al. recommend focusing on the importance of the IC discussion to improve its process [9]. Therefore, in the context of clinical trials, SDM is also expected to support high-quality IC conversations that will enable true participant comprehension and better alignment between enrolment decisions and participant values [19].

Accordingly, SDM might be useful to conduct ethical and patient-centred clinical trials. However, the perception of SDM among stakeholders in Japan remains unknown. Therefore, we focused on the perceptions of CRCs who routinely support the IC process to examine strategies for implementing or facilitating SDM in clinical trials.

## Methods

### Aim

This study aimed to (1) identify Japanese CRCs’ perception of the current status of SDM implementation and its influencing factors, and to (2) obtain suggestions to enhance the SDM process in clinical trials.

### Definition of the SDM

‘Decision-making’ in this study refers to ‘deciding whether to participate in a clinical trial’. Thus, we defined SDM as ‘a process in which the patient and the healthcare professional decide on participation or non-participation while sharing adequate information and respecting the patient’s values and preferences’.

### The SDM process in clinical trials

The Shared Decision-Making Questionnaire for Doctors (SDM-Q-Doc) is a tool developed to assess physicians’ perceptions of SDM implementation, comprising nine items with one factor and rated on a six-point Likert scale [20]. The Japanese version of the SDM-Q-Doc has good internal consistency (Cronbach’s alpha = 0.87) [21]. Therefore, we modified the Japanese version to include the following nine items:

- *Item 1* Clearly inform my patient that a decision (to participate or not in the clinical trial) must be made.
- *Item 2* Attempt to understand exactly how they want to be involved in decision-making.
- *Item 3* Inform my patient that they have the option of not participating in the clinical trial.
- *Item 4* Precisely explain to my patient the advantages and disadvantages of participating or not participating in the clinical trial.
- *Item 5* Help my patient understand all the information in the ICDs.
- *Item 6* Ask my patient whether they prefer to participate or not participate in the clinical trial.
- *Item 7* Thoroughly weigh the options of participation or non-participation with my patient.
- *Item 8* Select an option (to participate or not in the clinical trial) together with my patient.
- *Item 9* Reach an agreement with my patient on how to proceed.

### Study design

This study used a web questionnaire to record responses from a cross-sectional survey.

### Participants

The participants were CRCs working at Japanese medical institutions, including hospitals and clinics. The target sample size was estimated to be 350, based on the assumption that Japan has approximately 5000 CRCs within 5% tolerance, a confidence level of 95%, and a response ratio of 0.5.

The invitation letters were sent to the clinical trials support departments of 1,087 Japanese medical institutions, including hospitals and clinics, on 07 November, 2020. We selected medical institutions that were registered in the network of the Center for Clinical Trials, Japan Medical Association, and with publicly available addresses. No pre-test of the questionnaire was conducted prior to the survey.

Each envelope included a leaflet describing the SDM process in clinical trials and the QR-coded questionnaire. After reading these, CRCs who agreed to participate in this study answered the web-based questionnaire. There were no specific inclusion or exclusion criteria for participants. In addition, a gift card (worth approximately $5) was sent to those who requested it as an acknowledgement.

### Questionnaire development

#### Theoretical framework: Theory of Planned Behaviour

Integrating SDM into daily practice often requires behavioural change from health professionals [22]. The same might apply to the implementation of SDM in clinical trials. Therefore, to identify factors affecting the implementation of SDM by CRCs, we developed a questionnaire based on the Theory of Planned Behaviour (TPB).

TPB is a social cognitive theory often used to predict the behaviour of healthcare professionals [23]. It has been used in many studies examining the factors influencing SDM behaviour [22]. Therefore, we developed a questionnaire using TPB as a theoretical framework. According to the TPB, three constructs independently determine an individual’s intention to perform a particular behaviour. These are (1) attitude, the individual’s positive or negative evaluation of a specific behaviour; (2) subjective norms, the individual’s perceived social pressure from other individuals or groups about adopting a specific behaviour, and (3) perceived behavioural control, the individual’s perceived control ability to perform a specific behaviour [24].

In this study, CRC’s perception of the SDM implementation status was classified as an objective variable, ‘behavioural intention’. ‘Attitude toward SDM’, ‘subjective norms of CRC’s SDM implementation’, and ‘barriers to autonomous decision-making and difficult steps in the SDM process (perceived behavioural control)’ were classified as explanatory variables that affect behavioural intention.

#### The status of the SDM implementation (CS)

The participants were asked: ‘How often do you currently implement this step?’ for each of the nine steps and answered using a six-point scale from 1 (*not at all*) to 6 (*always*). The total score ranged from 9 to 54.

#### Attitude toward SDM (AT)

AT was measured by asking: ‘Do you think the SDM process is favourable for clinical trials?’ (1 = *strongly disagree* to 6 = *strongly agree*). Higher scores indicated a more positive attitude.

#### Subjective norms for CRC’s SDM implementation (SN)

The following stakeholders were considered normative for CRCs: Institutional Review Board (IRB) members, patients, patients’ families, investigators, and sponsors.

SN was measured by answering: ‘They (each stakeholder) would agree with CRCs’ SDM implementation’ (5 items), and ‘Do you follow their views?’ (5 items), using a six-point scale from 1 (*strongly disagree*) to 6 (*strongly agree*). Higher scores indicated stronger normative beliefs.

#### Perceived behavioural control

SDM is founded on the principle of respect for autonomy [25]. Thus, barriers to respect for autonomy can be barriers to SDM implementation. Therefore, an 11-item questionnaire was newly developed to assess perceptions of barriers to autonomous decision-making (BA) in clinical trials. This questionnaire was based on a Japanese study of difficulties encountered by nurses in the care of terminally ill cancer patients [26], a scale to measure nurses’ difficulty with cancer care [27], a study of work-related stress and anxiety experienced by Japanese CRCs, [28] and our previous qualitative study on decisional needs in clinical trials [29].

The participants answered the following questions on a scale of 1 (*strongly agree*) to 6 (*strongly disagree*). Total scores ranged from 11 to 66, with higher scores indicating having control of the SDM implementation:

1. It is difficult to explain clinical trials using ICDs.
2. Many patients do not understand the explanations of clinical trials.
3. Explanations by investigators are insufficient.
4. My explanation may not be sufficient.
5. I cannot answer patients’ questions adequately.
6. I am under pressure from physicians (investigators) to obtain IC.
7. I am under pressure from sponsors to obtain IC.
8. I sometimes think I am unable to support decision-making from a neutral standpoint.
9. I am aware of patients who decide to participate in clinical trials to respect the investigator’s intention.
10. I am aware of patients who decide to participate in clinical trials to respect their family’s intentions.
11. Sometimes, I think that patients’ wishes are not respected.

In addition, to identify difficulty within the nine steps of the SDM process, we asked participants to choose which steps they found difficult to implement (multiple choices allowed). These responses were scored from 1 to 9 points; the higher the number of choices, the lower the score (number of difficult steps in the SDM process; ND).

#### Socio-demographic characteristics

Socio-demographic information gathered included sex, age, qualification in the healthcare profession (nurse, pharmacist, clinical laboratory technician, other, or none), and years’ experience as a CRC.

### Reliability and validity of the measures

To evaluate the reliability and validity of CS, SN, and BA, exploratory factor analysis (Principal axis, Promax Rotation) was used to confirm the factor structure. Cronbach’s alpha was calculated to examine the internal consistency of the items in each measure. Confirmatory factor analysis (CFA) was conducted to examine the structural validity of the extracted factors in each measure. The comparative fit index (CFI) and the root mean squared approximation error (RMSEA) were used as model fit indices: a CFI value of 0.90 or higher is generally considered an acceptable model fit; an RMSEA value of less than 0.05 indicates a good fit, and a value of less than 0.08 is acceptable [30].

### Statistical analysis

Descriptive statistics, including mean, standard deviation (SD), frequencies, and percentages, were used to describe the participants’ socio-demographic characteristics and the variables. The relationships between the variables were analysed using Pearson’s correlation coefficient. Multiple regression analysis was conducted using AT, SN, BA, and ND as explanatory variables to identify factors affecting CS. Data analysis was performed using SPSS version 28.0 and Amos version 28.

## Results

### Participants’ characteristics

Between 7 December, 2020, and 7 January, 2021, 373 responses were received. Our invitation letters were sent to 1087 sites, and responses were received from CRCs at each medical institution; assuming a response from one person per site, the response rate was approximately 34.3%. As responses reached the target number of 350, no reminders were sent to the medical institutions.

No one was excluded due to missing values or incomplete responses.

Of the 373 participants in this study, 323 (86.6%) were female. The participants had a mean age of 42.4 years (SD = 9.915). More than 60% had less than 10 years of experience as a CRC. Most participants were healthcare professionals (nurses, pharmacists, medical laboratory scientists, and others), while 11.8% had no qualifications in the healthcare profession. (Table 1).

**Table 1:** Characteristics of study participants (*N* = 373)

	n (%)
Sex	
Male	50 (13.4)
Female	323 (86.6)
Age	
Mean/SD	42.4 (9.92)
Years’ experience as a CRC	
≦ 3	119 (31.9)
4–9	112 (30.0)
10≧	142 (38.1)
*Qualification*	
Nurse	175 (46.9)
Pharmacist	71 (19.0)
Medical laboratory	69 (18.5)
Scientist	
Other	14 (3.8)
None	44 (11.8)

### Reliability and validity of the measures

#### The current status of SDM implementation (CS)

Exploratory factor analysis detected two factors with an eigenvalue above one.

The first factor from the SDM-Q-Doc included items 1, 3, 4, 5, and 6 (see: The SDM process in clinical trials). We thus named Factor 1, ‘The steps performed by the health care professional concerning the patient’.

The second factor included items 2, 7, 8, and 9. Factor 2 was named, ‘The steps performed collaboratively by patients and health care professionals’.

Factor loadings for factor 1 were above 0.441, and factor 2 was above 0.424. To test the reliability of CS, Cronbach’s alpha coefficient was calculated, yielding values of 0.743 and 0.709 for factors 1 and 2, respectively. Cronbach’s alpha was 0.770 for the nine items overall. The validity of CS was confirmed using confirmatory factor analysis. The CFI was 0.919, and the RMSEA was 0.092. Some error covariances were observed between items (items 2 and 4, items 3 with 1 and 4, and items 8 and 9).

#### Subjective norms for CRC’s SDM implementation (SN)

Exploratory factor analysis detected two factors with an eigenvalue above one: ‘Stakeholders’ agreement with CRCs’ implementation of SDM’ and ‘CRCs’ perception that stakeholder views should be followed’. Factor loadings for each factor were above 0.398 and 0.470, respectively. Cronbach’s alpha coefficients of the two factors were 0.800 and 0.737, respectively, and 0.796 for the 10 items overall.

The validity of SN was confirmed using confirmatory factor analysis. The CFI was 0.957, and the RMSEA was 0.081. Some error covariances were observed between ‘patients’ agreement with SDM’ and ‘CRCs’ perception that should follow patients’ view’, and items regarding ‘IRB, patient families, and investigator agreement with SDM’.

#### Perceived barriers to autonomous decision-making (BA)

Exploratory factor analysis detected four factors with an eigenvalue above one. Based on these results, each domain was entitled: Factor 1 (#1–3) (‘Insufficient resources to facilitate patients’ understanding’), Factor 2 (#4 and 5) (‘Lack of decision support skills’), Factor 3 (#6 and 7) (‘Pressure to obtain IC’), and Factor 4 (#8–11) (‘Relational barrier’). Factor loadings for each factor were above 0.576, 0.760, 0.449, and 0.366 respectively. Cronbach’s alpha coefficients of the four factors were 0.673, 0.810, 0.550, and 0.628, respectively, and 0.755 for the 11 items overall. The validity of BA was confirmed using confirmatory factor analysis. The CFI was 0.905, and the RMSEA was 0.081.

### The current status of SDM implementation and difficult SDM steps

#### The current status of SDM implementation

No participants answered ‘*not at all*’ (score of 1) for item 3, ‘Inform my patient that they have the option of not participating in the clinical trial’; item 4, ‘Precisely explain to my patient the advantages and disadvantages of participating or not participating in the clinical trial’; item 5, ‘Help my patient understand all the information in the ICDs’; and item 6, ‘Ask my patient whether they prefer to participate or not in the clinical trial’. More than half of the CRCs responded ‘*a little*’ or ‘*always*’ (score of 3 or higher) to all steps in the SDM process. However, scores for item 2, ‘Attempt to understand exactly how they want to be involved in decision-making’ (72.7%); item 7, ‘Thoroughly weigh the options of participation or non-participation with my patient’ (73.2%); and item 8, ‘Select an option (participate or not in the clinical trial) together with my patient’ (59.5%) were relatively low. (Table 2).

**Table 2:** The current status of SDM implementation and difficult SDM steps (*N* = 373)

Factor	Items#	The current status of SDM implementation			Frequency (%) of answering ‘difficult’ in each step
		Mean (SD)	Max–Min	Score of 3 or higher (%)	
	1	5.03 (1.062)	1–6	92.5	35 (9.4)
	3	5.79 (0.541)	3–6	99.2	12 (3.2)
1	4	5.47 (0.727)	2–6	98.7	90 (24.1)
	5	5.49 (0.721)	2–6	98.4	73 (19.6)
	6	5.48 (0.847)	2–6	96.8	26 (7.0)
	2	4.21 (1.355)	1–6	72.7	172 (46.1)
2	7	4.21 (1.265)	1–6	73.2	216 (57.9)
	8	3.86 (1.525)	1–6	59.5	144 (38.6)
	9	4.89 (1.121)	1–6	89.5	73 (19.6)
	Total score	44.42 (5.690)	28–54	–	–-

Factor 1: The items performed by the health care professional concerning the patient. Factor 2: The items performed collaboratively by patients and health care professionals

#### Difficult SDM steps

The step that most CRCs rated as ‘difficult’ was item 7 (57.9%), followed by item 2 (46.1%), item 8 (38.6%), and item 4 (24.1%).

These were consistent with the items with relatively low CS scores, except for item 4.

Conversely, item 3 (3.2%) was reported as the least difficult (Table 2).

Moreover, 29.8% of participants answered that all nine items were difficult. The most frequent quantity of difficult items was eight (35.4%). Contrarily, 1.1% of participants selected ‘zero’ items as difficult. These responses were scored inversely in a range of 1–9 points. Thus, the higher the number of choices, the lower the score, and the mean was 2.25 (SD = 1.256) (Table 3).

**Table 3:** The number of difficult items in the SDM process (*N* = 373)

The number of choices	Score	Frequency (%)
1	9	111 (29.8)
2	8	132 (35.4)
3	7	84 (22.5)
4	6	33 (8.8)
5	5	8 (2.1)
6	4	1 (0.3)
7	3	0
8	2	0
9	1	4 (1.1)

### Attitude toward SDM

No participant answered ‘strongly disagree’ to the question, ‘Do you think the SDM process is favourable for clinical trials?’. Ninety-four percent of the participants had a score of 3 or higher (mean score = 4.89, SD = 0.914), indicating a positive attitude toward the SDM process.

### The subjective norms for the SDM implementation of CRCs

The overall score range for the responses was 27–60 (mean score = 44.0, SD = 6.39), indicating that participants tended to be highly normative. Responses to the five items on the ‘Stakeholders’ agreement with CRCs’ implementation of SDM (Factor 1)’ scale ranged from 5 to 30 (mean score = 20.5, SD = 4.05). The 5 items on the ‘CRCs’ perception that stakeholder views should be followed (Factor 2)’ ranged from 14 to 30 (mean score = 20.5, SD = 4.05) (Table 4).

**Table 4:** Subjective norms for the SDM implementation (*N* = 373)

	Max–min	Mean (SD)
Factor 1: Stakeholders would agree with CRCs’ SDM implementation		
Factor 2: Do you follow their views?		
IRB members	1–6	4.15 (1.14)
	1–6	4.60 (1.10)
Patients	1–6	4.35 (1.03)
	3–6	5.46 (0.82)
Patients’ families	1–6	4.41 (1.02)
	2–6	5.26 (0.88)
Investigators	1–6	3.84 (1.08)
	1–6	4.63 (1.10)
Sponsors	1–6	3.80 (1.16)
	1–6	3.94 (1.34)
Factor 1	5–30	20.5(4.05)
Factor 2	14–30	20.5 (4.05)
Total	27–60	44.0 (6.39)

### Perceived barriers to autonomous decision-making (BA)

**Fig. 1:**
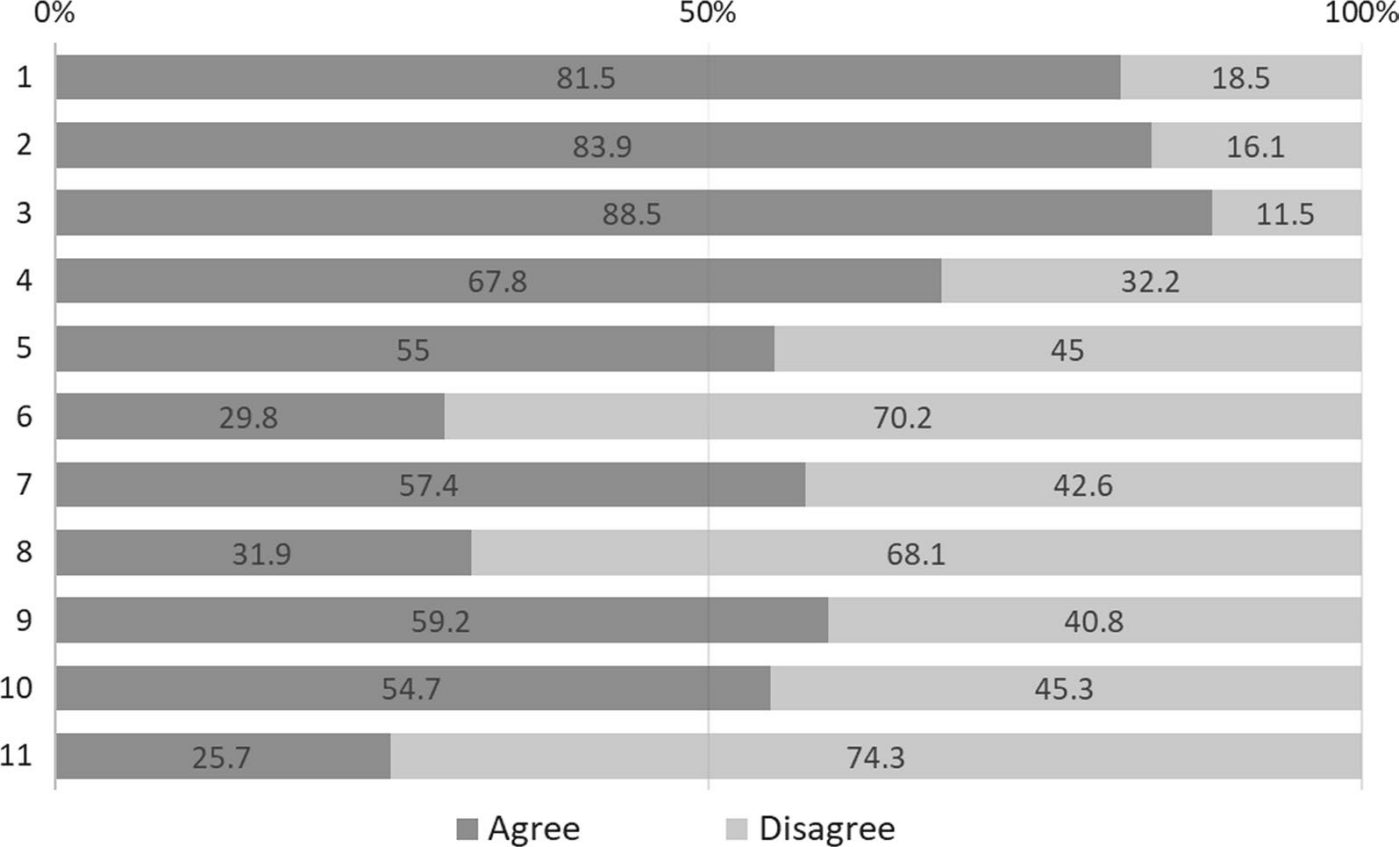
Perceived barriers to autonomous decision-making: percentages of ‘Agree’ and ‘Disagree’ (*N* = 373). Agree: score 1–3, Disagree: score 4–6

Table 5 shows the mean scores (SD) for each item. Responses were then divided into ‘agree’ and ‘disagree’. Figure 1 shows the percentages. More than 80% of CRCs identified the items of Factor 1 (#1–3) as barriers. Relatively low ‘agree’ responses were recorded for #11 (25.7%), #6 (29.7%), and #8 (31.9%), which were included in Factors 3 and 4.

**Table 5:** Means (SD) of perceived barriers to autonomous decision-making (*N* = 373)

Factor	Item#	Mean (SD)	Agree (%)
1	1	2.49 (1.18)	81.5
	2	2.52 (1.05)	83.9
	3	2.29 (1.06)	88.5
2	4	3.0 (1.19)	67.9
	5	3.37 (1.27)	54.9
3	6	4.27 (1.49)	29.7
	7	3.23 (1.66)	57.4
4	8	4.09 (1.33)	31.9
	9	3.36 (1.20)	59.3
	10	3.43 (1.18)	54.7
	11	4.25 (1.15)	25.7
	Total score	36.3 (7.50)	–

Factor 1: Insufficient resources to facilitate patients’ understanding. Factor 2: Lack of decision support skills. Factor 3: Pressure to obtain IC. Factor 4: Relational barrier

### The relationships between the variables

The relationships between the total scores of CS, AT, SN, BA, and ND were assessed by computing Pearson’s correlation coefficient. Table 6 shows the results. CS was related positively to AT (*r* = 0.180,* p* < 0.001), SN (r = 0.148, p = 0.004), BA (*r* = 0.207, *p* < 0.001), and ND (*r* = 0.192,* p* < 0.001). Moreover, significant positive correlations were observed between AT and SN (*r* = 0.334,* p* < 0.001), but AT was negatively related to BA (*r* = − 0.128, *p* = 0.013).

**Table 6:** The relationships between the variables; Pearson’s correlation analysis

	CS	AT	SN	BA	ND
AT	.180***				
SN	.148**	.343***			
BA	.207***	− .128*	− .084		
ND	.192***	− .071	− .079	.237***	

CS, The current status of SDM implementation; AT, Attitude toward SDM; SN, Subjective norms for the SDM implementation of CRCs; ND, The number of difficult steps in the SDM process; BA, Perceived barriers to autonomous decision-making; statistically significant: *p = .013, **p = .004, ***p < .001

### Factors affecting the current status of SDM implementation

Based on the above, for the multiple linear regression analysis, the total score of CS was set as the objective variable, and the answers to questions on the perceptions of the SDM process (AT, BA, SN, and ND) were set as explanatory variables. The results showed that *R*^2^ = 0.140 and adjusted *R*^2^ = 0.128, *p* < 0.001 for this model.

The variables that significantly influenced CS were AT (*t* = 3.400, *p* < 0.001), SN (*t* = 2.239, *p* = 0.026), BA (*t* = 3.957, *p* < 0.001), and ND (*t* = 3.317, *p* = 0.001) (Table 7).

**Table 7:** Factors affecting CS: multiple linear regression analysis (*N* = 373)

Variable	B	β	*t*	*p*	95% CI
AT	1.107	0.178	3.400	< .001*	[0.467, 1.748]
SN	0.104	0.117	2.239	.026*	[0.013, 0.195]
BA	0.152	0.200	3.957	< .001*	[0.076, 0.228]
ND	0.757	0.167	3.317	.001*	[0.308, 1.205]

*R* (multiple regression) = .351; Adjusted *R*^2^ = .123. *statistically significant (*p* < .05). Cl, Confidence interval; CS, The current status of SDM implementation; AT, Attitude toward SDM; SN, Subjective norms for the SDM implementation of CRCs; ND, The number of difficult steps in the SDM process; BA, Perceived barriers to autonomous decision-making

## Discussion

SDM presumably occurs between physicians and patients; thus, most research has focused on physicians. However, other healthcare professionals, like nurses, have recently been involved in decision-making and play a supportive role in helping patients form preferences and discuss the pros and cons of treatment options [30]. In the context of clinical trials, CRCs are involved in patients’ decisions to participate in clinical trials in Japan. This study thus focused on their perceptions.

This study showed that most CRCs had positive perceptions of SDM and believed that they were already implementing it. The explanatory variables based on TPB were correlated with positive attitudes towards the SDM, high normative beliefs, and perceived behavioural control for SDM, significantly influencing the perception of the SDM implementation status among the CRCs.

First, Godin et al. described that moral values significantly impact intention. They reasoned that people’s sense of personal obligation to perform a behaviour would influence the motivational force of intention [24]. A systematic review of behavioural intentions in SDM also reported that subjective norms were significantly associated with intentions in many studies [25].

Consistent with previous studies, the results of this study convey a positive correlation between SN and CS and between SN and AT. A subjective norm refers to how an individual’s immediate environment influences their actions. Thus, in the case of SDM, this construct can refer to the person’s peers, mentors, or licensing bodies, and the influence of the patient [22]. This is especially because CRCs are expected to behave ethically by IRB members, patients, patient’s families, investigators, and sponsors. Subjective norms are also considered the most frequent determinant of intention due to their influence on interpersonal relationships [22]. Therefore, it is likely that CRCs’ normative beliefs on AT and CS were positively influenced by regulatory compliance requirements, ethics, and relationships with stakeholders.

A behaviour’s perceived ease or difficulty is referred to as perceived behavioural control. If an individual’s attitude and subjective norms regarding a behaviour are more positive, then the intention to engage in that behaviour will be increased [24]. This is because they have control over their behaviour. A systemic review by Thompson-Leduc et al. found that perceived behavioural control significantly influenced the intention to implement SDM in half of the studies analysed [22].

Consistent with previous studies, our multiple regression analysis revealed that perceived behavioural control (ND and BA) had a significant positive correlation and effect on CS. However, the descriptive statistical results of ND and BA showed that many CRCs identified the SDM process as difficult, with many barriers. In the context of clinical trials, SDM is still a new concept; thus, there is a lack of related knowledge and acquired skills among clinical trial stakeholders, including CRCs. Additionally, the significant negative correlation between BA and AT may also reflect a lack of consensus regarding the characteristics of the target population (those considering participating in clinical trials), as described by Gravel et al. [31].

Therefore, attempting to implement SDM without adequate knowledge and skills and relying only on normative beliefs may be problematic. The systematic review by Thompson-Leduc et al. highlighted that a lack of self-efficacy and expertise in SDM limits its impact. They suggested that, to better implement SDM in clinical settings, providing training activities to healthcare professionals is critical [22]. In an updated review, researchers contended that these barriers still exist, and gaps in knowledge continue to affect SDM implementation and should be further studied [32].

Given the findings of this study, providing appropriate training on SDM to CRCs could enable the facilitation of SDM based on correct knowledge and understanding. The results of BA and ND could be useful when developing training programs. For example, programmes could focus on SDM steps and decision support skills, which many CRCs find difficult.

In addition, CRCs are expected by sponsors and investigators to recruit patients within a certain timeframe. Nonetheless, the result of factor 3 in the BA (item 6, ‘I am under pressure from physicians to obtain IC’; item 7, ‘I am under pressure from sponsors to obtain IC’) also showed that CRCs are under pressure to obtain consent. Therefore, efforts to increase the awareness of SDM among clinical trial stakeholders, who exert a normative influence on CRCs, would also be effective. That is, the understanding of SDM by stakeholders who influence CRCs, especially investigators and sponsors, would be essential for the successful implementation of authentic SDM in clinical trials.

### Limitations

This study has several limitations. First, the sample of CRCs recruited might have already had an interest in decision support. As participants who agreed to participate in the research received a leaflet regarding SDM, we should consider the possibility of volunteer or social desirability bias. These would have influenced positive perceptions of SDM and normative responses.

Additionally, the low response rate and the small size of the dataset might affect the statistical significance of the results. The absence of reminders may lead to selection bias and non-response bias, undermining the representativeness of the population in the study results.

Although most CRCs in this study believed that they were already implementing SDM, further research, such as mixed-methods studies that include interviews with participants through audio and/or video-recording conversations and analysing them, or surveys targeting patients, might be necessary to deepen the interpretation of this study’s results. In this study, the SDM-Q-Doc was used, and a survey using the 9-item Shared Decision-Making Questionnaire [4] to understand CRC’s actual implementation of SDM from the perspective of patients participating in clinical trials would be useful. Furthermore, In Japan, the stakeholders’ perceptions of SDM as influencing CRC normative behaviour remain unclear; this is a future research avenue for the promotion of SDM.

The validity of the measures used in this study also must consider the limitations of interpretation. In particular, the assumption of covariates between some errors led to acceptable model fits for CS and BA. This indicates that they may have measured overlapping concepts across items. In addition, CS used a modified version of the SDM-Q-Doc, which originally had a one-factor structure [21], whereas a two-factor structure was developed in this study. This may be a result of modifying the measure for CRCs rather than utilising it for physicians.

Finally, in this study, each item of CS was unweighted and included equally in the total score. SDM in clinical trials may involve concepts not fully explored in treatment choice decision-making. Weighting variables in SDM in clinical trials are important issues to be explored in future studies.

## Conclusions

In this study, we examined the perceptions of Japanese CRCs regarding the current state of SDM implementation and its influencing factors. Many CRCs in the study perceived that they were already implementing SDM. Normative beliefs, perceived behavioural control, and positive attitudes towards SDM were also found to positively influence perceptions of SDM implementation. However, the results of this study indicate a lack of knowledge of SDM and a lack of decision-making support skills among CRCs. SDM implementation might be less successful when attempts are made to facilitate it without sufficient knowledge and skills and by solely relying on normative beliefs. Therefore, providing appropriate training on SDM to CRCs and raising awareness among stakeholders could improve the facilitation of SDM.

## Data Availability

The data that support the findings of this study are available on request from the corresponding author, MF.

## Abbreviations

AT: Attitude toward shared decision-making
BA: Perceived barriers to autonomous decision-making
CFA: Confirmatory factor analysis
CFI: Comparative fit index
CI: Confidence interval
CRC: Clinical research coordinator
CS: The current status of shared decision-making implementation
IC: Informed consent
ICD: Informed consent document
IRB: International Review Board
ND: Number of difficult steps in the shared decision-making process
RMSEA: Root mean squared approximation error
SD: Standard deviation
SDM: Shared decision-making
SDM-Q-Doc: Shared Decision-Making Questionnaire for Doctors
SN: Subjective norms for the shared decision-making implementation of clinical research coordinators
